# Factor Analysis of EMA-Scale on Adolescent Adjustment From a Developmental Perspective: A Short Form

**DOI:** 10.3389/fpsyg.2018.02406

**Published:** 2018-12-03

**Authors:** Lucía Jiménez, Susana Menéndez, Victoria Hidalgo

**Affiliations:** ^1^Department of Developmental and Educational Psychology, University of Seville, Seville, Spain; ^2^Department of Social, Developmental and Educational Psychology, University of Huelva, Huelva, Spain

**Keywords:** adolescent adjustment, validation, factor analysis, positive development, secondary education

## Abstract

Many published instruments for assessing adolescent adjustment can be implemented in the school context. However, most of them fail to include a comprehensive and positive theoretical perspective of adolescent development and, even when they do, priority is often given to the clinical perspective, or problems with ecological validity and cost-effectiveness emerge. The Magallanes Adaptation Scale is a 90-item Likert-instrument designed for Spanish-speaking adolescents in order to screen several adjustment areas from a holistic and positive perspective of development. Although some evidence of its psychometric robustness has been tested, no confirmatory analysis of its structure has been published. This paper analyzes the items and the factor structure (exploratory factor analysis and confirmatory factor analysis, using the split-half method) of the scales. Participants were 948 Spanish adolescents (49.84% girls) aged between 11 and 17 and stratified sampled. Thirty-six items were removed from the item analysis. The results of the exploratory factor analysis revealed five factors, excluding mother's adaptation. Several models were tested during the confirmatory factor analyses, with a 24-item second-order four-factor solution being found to have the best adjustment indicators. The short version proposed in this paper can constitute a helpful tool with screening purposes to help school teachers to assess students' overall development beyond mere academic performance, although further validity research is needed.

## Introduction

Formal educational systems have evolved from an industrialization project focusing on literacy, to a more encompassing view of education aimed at promoting child development from an integrated perspective (Delors et al., 1996). Formal education in knowledge society needs to promote minds able to understand and transform a global world (OEI, 2010). This means that besides “learning to know,” schools should prepare children and adolescents for “learning to do,” “learning to be”, and “learning to live together” (Delors et al., 1996). Consequently, there is a wide consensus about not just academic, but also professional and civic competences need to be integrated into the school curriculum (Ortega, 2005; Hartley and Soo, 2009). In this arena, those competences related to social cognition adquires a salient role (Herschbach, 2012; Brizio et al., 2015).

This integrated view of formal education is in consonance with the current perspective on adolescent development. From developmental psychology, development is nowadays described from both comprehensive and organizational assumptions. A comprehensive or organizational view on development is based on the idea that children and adolescents constitute dynamic and integrated systems in which all development domains mutually interact (Wenar and Kerig, 2000). Consequently, child and adolescent performance is a multi-dimensional process in which the adaptive or non-adaptive resolution of developmental tasks influences other developmental domains. Considering school as a developmental context, we count on empirical evidence supporting this assumption. For example, behavioral problems have been negatively related to academic competence and classroom adjustment (Lane, 2003; Smokowski et al., 2004; Frazier et al., 2007), and social skills and social performance have been linked to academic competence and school behavior (Dishion and Piehler, 2007; Wentzel and Looney, 2007). Alongside this organizational perspective of development, probabilistic but not deterministic principles should also be considered (Cicchetti and Toth, 1997).

In addition to a comprehensive or organizational perspective, developmental contextualism suggest to regard the whole pattern of dynamic person-contexts interactions as the key phenomenon of psychological development (Lerner and Steinberg, 2004). This perspective is particularly important during adolescence, which is a transition period from both an educational and a developmental point of view. Importance of person-context fit is heightened during this period due to the normative physical and psychological changes undergone by adolescents (Lerner and Steinberg, 2004), as well as the educational challenges they are faced with in high-school (e.g., diversification of teachers, departmental organization, fluctuation in their peer group and larger schools) (Eccles and Roeser, 2003). It should be highlighted that, despite the named vulnerability, adolescent development is nowadays conceptualized from a positive perspective aimed at promoting adolescents' social, cognitive and emotional competencies (Oliva et al., 2010).

The developmental contextualism has led to consider adaptation to school context as a key developmental task in adolescence. The integrated view of formal education, together with an organizational and positive comprehension of development, has translated school adaptation into a multi-domain adjustment construct that encompasses not only academic, but individual and social performance (Flammer and Alsaker, 2006; Wentzel and Looney, 2007). We do not count on one single model that guides what healthy school adjustment entails, but besides academic achievement, social competence has been emphasized (Wentzel, 2003). On this regard there is a wide scope of dimensions that have been examined, although peers interactions, relationships with teachers, and belonging with the school stand out (e.g., Zettergren, 2003; Murdock and Bolch, 2005; Määttä et al., 2007; Oramah, 2014). Even for these components, diverse indicators have been described in scientific literature: for example, frequency of interactions, satisfaction or perceived support concerning relationships with peers and teachers; as well as knowledge and respect from school institution, acceptation of norms and values, or attachment. Despite this diversity, a person-environment fit approach remains. Thus, both individual and contextual influences are considered from an interactive framework (Wentzel, 2003). As a result, current integrated views of adolescent adjustment from school context also take into consideration individual, family, and community influences on adjustment (Eccles and Roeser, 2003; Eccles, 2004; Dowrick and Crespo, 2005; Jiménez et al., 2009).

Within this framework, an integrated and contextualist view of adolescent adjustment from school context requires a comprehensive analysis of different facets. As a conclusion, teachers are required to focus not just on students' academic progress but also on other domains (Pozo and Pérez, 2009). To do this, teachers need assessment instruments to provide them with a holistic view of students' progress and development, beyond mere academic performance. To this purpose, there are several instruments for assessing students' performance from a developmental perspective, although concerns have been raised regarding their developmental sensitivity, their comprehensive perspective and the ability to capture person-context interactions (Jiménez et al., 2013). This paper analyzes, from a psychometric viewpoint, a Spanish measure of adolescent adjustment to be applied at school context from a positive, integrated, and contextualist developmental perspective.

There are many published instruments for assessing adolescent adjustment that can be implemented in the school context. In accordance with the classic view of formal education, there are many measures that focus on academic variables such as school performance or intelligence (e.g., Schaefer and Edgerton, 1978; Wechsler Psychological Corporation, 2003), and while several instruments do include a more comprehensive view, a clinical perspective not appropriate for the school context tends to dominate (e.g., Achenbach, 1991). A few measures designed specifically for the school context remain but they usually include a large number of questions, while screening options are not always available (e.g., Wingenfeld et al., 1998). Moreover, sophisticated and expensive computer procedures are sometimes required (e.g., Reynolds and Kamphaus, 2004), making it harder for teachers to administer them for screening purposes. In relation to ecological validity, most of these instruments have been tested with American samples (e.g., Gresham and Elliot, 1990), despite cultural disparities with Spain.

The Magallanes system of assessment developed in the Spanish context that offer several insturments aimed at assessing child and adolescent adjustment (i.e., anxiety, impulsivity, attention an so on). Specifically, the *Escalas Magallanes de Adaptación* [Magallanes Adaptation Scale] (*EMA*, García and Magaz, 1998) is a specific instrument that defines adaptation as those developmental processes that allow adolescents to adjust their behavior both to their own needs as well as contextual requirements. Therefore, the *EMA* reflects a holistic view of adolescent adjustment based on a developmental approach. This measure takes into account a comprehensive view of adaptation at school (at an academic, institutional, and social level), and also includes other indicators that have been pointed out in literature because its influence on adjustment at school (personal and family-related). The *EMA* refers to different adaptation areas relevant to adolescents, and is congruent with a positive development framework as includes positive indicators of performance and adaptation (Brooks et al., 2012). Moreover, this instrument is respectful with an interactive perspective of adolescent development, as includes indicators concerning student's behavior, as well as contextual influences. This measure offers relevant information at a screening and descriptive level with a Spanish-speaking population both at scale (Aragón and Bosques, 2012) and item level (Suárez et al., 2012).

The manual for the *EMA* offers evidence of its psychometric robustness (García and Magaz, 1998), although no confirmatory analysis of its structure has yet been published by either the original authors or others. However empirical validations of other Magallanes instruments have recently been published with Spanish-speaking populations, including reliability, and validity testing (Servera et al., 2009), and dimensionality analysis (Sandín et al., 2005; Martínez-Monteagudo et al., 2013), with satisfactory results. Rating information for a Spanish-speaking population has also been reported (Ison and Carrada, 2011).

In order to provide evidence of the psychometric soundness of the *EMA*, this paper analyzes *EMA* both at item and scale level (exploratory factor analysis and confirmatory factor analysis, with the split-half method).

## Materials and methods

### Participants

Nine hundred and forty-eight adolescents from the general population in southern Spain (Seville) participated in the study. The sample was equally distributed by gender (50.16% boys and 49.84% girls) and ages ranged from 11 to 17 years old (*M* = 12.88, *SD* = 1.41). Of the total group, 19.57% of participants received educational support at school. Most lived with both parents (92.72%), although there was a high degree of variability concerning parents' educational level: 37.08% of parents were illiterate or had attended primary school, 38.76% had completed high school and 24.16% had a university degree.

### Measures

The *EMA* (García and Magaz, 1998) instrument is a self-administered test that evaluates adolescent adjustment at school in several domains. This measure includes several indicators for school adaptation (teacher-related, peers-related, and to the school as an institution) and it considers individual and family influences. It is designed for helping school teachers to screen the level of adaptation to the following domains: adaptation to teachers (14 items), to peers (11 items), to the school as an institution (6 items), to the mother (20 items), to the father (20 items), and personal adaptation (19 items). Teachers- and peers-related adaptation refer to the frequency of the interaction, the satisfaction with the relationship, and the perceived support (e.g., “*I talk with my teachers/classmates*,” “*I like my teachers/classmates*,” “*My teachers/classmates support me if I ask for help*”). Adaptation to the school as an institution includes commitment with school values and norms, as well as satisfaction (e.g., “*Even I dislike school rules, I comply with them*,” “*I feel satisfy with my school*”). Mother- and father-related adaptation refer to the quality of the interaction, as well as the perceived support (e.g., “*I listen to my mother/father, when she/he talks to me*,” “*My mother/father take an interest on my concerns”*). Personal adaptation refers to internalization issues (e.g., “*I feel sad and boring”*) and scores from this domain need to be reversed. In total, it comprises 90 items rated on a five-point Likert-type scale (from 0 = *never* to 4 = *always*) and takes about 15 min to complete. This instrument is a tool that can be used by school teachers for diverse purposes. For example, the *EMA* can be applied in order to identify vulnerable domains of adjustment, as an initial evaluation, or as progress indicator of students' adjustment at school in aforementioned areas.

### Data collection procedure

Within the framework of a larger research project, a stratified sampling procedure was followed taking into account the different districts of Seville (southern Spain). Thus, a number of schools was selected from each district according to their size in terms of population (*n* = 21 districts). The result was a representative sample of schools in Seville, including both public and private schools (73.84 and 26.16%, respectively). The management teams at the schools were contacted and asked to participate in the study, with an agreement rate of 100%. Respondents were selected from the following year groups within the Spanish education system: the final 2 years of primary school (28.37% of the sample), the first (63.01%) and second 2 years of secondary school (6.54%), and both years of the Baccalaureate (2.00%). Management teams asked for written and informed consent from the parents/legal guardians of the participants. Two researchers collected the data in a self-administered format. Every informant participated in the study voluntarily, after signing an informed consent form in accordance with the Declaration of Helsinki. The aims of the research project were explained and all participants were assured that their anonymity would be protected. Ethics approval was obtained from the ethics committee of the Andalusian Government.

### Data analysis

First, preliminary analyses were performed in order to check assumptions for linearity (through inspection of scatterplots among pairs of variables), non-multicollinearity and non-singularity (SMC < 1) at item level (Tabachnick and Fidell, 2007). The possible influence of both univariate and multivariate outliers was examined attending to the inter-quartile and Mahalanobis distance, respectively, (Tabachnick and Fidell, 2007).

Second, a double process of item analysis was conducted using SPSS vs. 20, with the aim of selecting items that would maximize the variance in the instrument and correlate highly with the true score of the latent variable (DeVellis, 2003). First, optimum difficulty was examined through the following criteria: (a) mean between 1.5 and 3.5, (b) *SD* above 0.5, (c) minimum range of 4, and (d) skewness and kurtosis between ±1 and ±2, respectively, (Ferrando and Anguiano-Carrasco, 2010). Second, internally consistent items were retained through significant positive corrected item-total correlations (>0.35), along with the absence of variation in reliability if the item was deleted (Barbero et al., 2006; Hair et al., 2008).

Third, in order to examine the factorial structure of the scale, we developed a combination of exploratory (EFA) and confirmatory (CFA) factor analyses using the two-half method (Floyd and Widaman, 1995). To do so, the sample was randomly split into two equivalent halves. Equivalence between the two halves was probed as no significant differences were found regarding child's gender, age, and parents' educational level. Moreover, similar distribution on grade level and type of center (public/private) was tested.

With the first half (*n* = 474), an EFA was conducted using the statistical package FACTOR vs. 9.2 (Lorenzo-Seva and Ferrando, 2006). Due to the ordinal nature of the items, the polychoric correlations matrix was used (Elosua and Zumbo, 2008). The Unweighted Least Squares method was chosen for factor extraction and the oblique Promin rotation method was used to increase interpretability, given the expected relationship between the underlying matrix factors. The EFA was developed following the recommendations of Ferrando and Anguiano-Carrasco (2010). Therefore, Timmerman and Lorenzo-Seva's (2011) parallel analysis (that combines Kaiser criteria and scree-plot test) was conducted for factor retaining decision. Moreover, the following criteria were considered for excluding items: (a) loadings lower than 0.63; (b) similar loadings in two or more factors (< 0.10); (c) substantive conceptual incoherence intra-factor; (d) excessively low communalities (< 0.20); and (e) item-total-correlation lower than 0.30 (Comrey and Lee, 1992; Tabachnick and Fidell, 2007). A minimum of three items per factor was also required (Stevens, 2002). In order to validate the correlation matrix structure, we conducted Bartlett's sphericity test (adequate if *p* < 0.05) and calculated the Kaiser-Meyer-Olkin (good if KMO >0.60) (Tabachnick and Fidell, 2007). Ordinal alpha by factor (>0.70) and McDonald's Omega for the total scale (>0.70) were examined (Elosua and Zumbo, 2008; Ferrando and Anguiano-Carrasco, 2010).

With the second half (*n* = 474), we performed several CFA using EQS vs. 6.1 (Bentler and Wu, 2002), following both statistical and theoretical parameters. Following the same strategy than for the EFA, for the computed CFAs the polychoric correlations matrix was used taking into account the ordinal nature of the variables. The Maximum Likelihood Estimation method was used with robust statistics (MLR), as a preferred method for normality violations (Mardia > 5 according to Bentler, 2006) with medium-size samples (Satorra and Bentler, 2001; DiStefano and Hess, 2005). Robust tests for standard errors were considered (Bentler, 2006). Several goodness-of-fit indexes were examined: Satorra-Bentler χ/fd statistic (< 5), NNFI and CFI indexes (values above 0.90 indicate an adequate model fit), and RMSEA index (with values of ≤ 0.06 indicating good model fit, around 0.08 indicating adequate fit, and ≥0.10 a poor fit) (Hu and Bentler, 1999; Barrett, 2007; Hair et al., 2008). As several rival models were tested with the CFA strategy, we performed a significance test on the difference between Satorra-Bentler scaled chi square statistics (Satorra and Bentler, 2001; Crawford and Henry, 2003). For the best model, statistical contribution and regression coefficients of the variables (Hair et al., 2008), as well as standardized errors were examined (Batista and Coenders, 2000) in order to refine the final solution. The Heise and Bohrnstedt's (1970) Omega coefficient was examined.

## Results

We probed normality, linearity, non-multicollinearity, and non-singularity assumptions with satisfactory results, identifying any univariate or multivariate outliers. Item analysis is summarized in Table 1. This procedure led us to retain those items with high discrimination power, high SDs, average scores around the medium point of the scale, and low skewness and kurtosis. Considering the criteria described in the method section, we excluded 34 items from subsequent analyses. Due to low corrected item-total correlations and an increasing in reliability if the item was deleted, three items were consequently removed. The items retained guaranteed that latent variables were preserved from this analysis.

**Table 1:** Descriptives about optimum difficulty and internally consistency at item level.

**Scale**	**Item**	***M***	***SD***	**Skewness**	**Kurtosis**	**Corrected item-total correlation**	**α if item deleted**
Teacher-related adaptation (α = 0.921)	1	2.90	1.03	−0.64	−0.45	0.65	0.914
	**2**	2.77	1.16	−0.68	−0.47	0.43	**0.921**
	3	2.70	1.10	−0.47	−0.66	0.70	0.912
	4	2.20	1.17	−0.05	−0.95	0.68	0.913
	5	2.20	1.99	−0.18	−0.92	0.75	0.910
	6	3.11	1.03	−0.99	0.17	0.58	0.916
	7	2.54	1.15	−0.31	−0.88	0.73	0.911
	8	2.24	1.16	−0.20	−0.83	0.73	0.911
	9	2.22	1.16	−0.22	−0.84	0.68	0.913
	10	1.96	1.25	0.06	−1.09	0.65	0.914
	11	1.89	1.26	0.15	−1.06	0.65	0.914
	12	2.31	1.17	−0.08	−1.08	0.52	0.919
	13	2.44	1.21	−0.18	−0.88	0.68	0.913
	14	2.35	1.14	−0.07	−1.08	0.58	0.916
Peer-related adaptation (α = 0.888)	**15**	3.26	0.95	–**1.17**	0.47	–	–
	16	2.75	1.04	−0.66	−0.09	0.72	0.867
	**17**	3.32	0.88	–**1.18**	0.54	–	–
	18	3.08	0.96	−0.83	−0.09	0.72	0.867
	19	2.52	1.21	−0.48	−0.71	0.65	0.878
	20	2.89	1.09	−0.77	−0.29	0.68	0.872
	21	2.62	1.04	−0.45	−0.42	0.73	0.866
	**22**	**3.61**	0.76	–**2.03**	**3.66**	–	–
	**23**	3.49	0.81	–**1.60**	1.87	–	–
	24	3.16	0.85	−0.81	0.16	0.60	0.881
	25	3.22	0.92	−1.09	0.68	0.70	0.871
School-as-an-institution-related adaptation (α = 0.875)	26	2.56	1.05	−0.35	−0.63	0.75	0.828
	**27**	2.90	1.18	−0.86	−0.24	0.50	**0.875**
	28	2.84	1.10	−0.61	−0.63	0.66	0.843
	29	2.89	1.15	−0.80	−0.37	0.64	0.848
	30	2.75	1.05	−0.53	−0.58	0.80	0.820
	31	3.05	1.01	−0.88	0.02	0.66	0.844
Mother-related adaptation (α = 0.803)	32	2.17	1.11	−0.17	−0.78	0.47	0.792
	**33**	**3.52**	0.80	–**1.67**	**2.21**	–	–
	**34**	3.17	1.06	–**1.23**	0.75	–	–
	35	2.98	1.06	−0.88	0.03	0.54	0.778
	36	3.29	0.89	−1.09	0.35	0.45	0.792
	**37**	3.45	0.81	–**1.59**	**2.41**	–	–
	**38**	**3.51**	0.78	–**1.64**	**2.27**	–	–
	**39**	**3.75**	0.58	–**2.72**	**8.02**	–	–
	**40**	3.37	0.90	–**1.51**	1.86	–	–
	41	2.62	0.97	−0.45	−0.21	0.63	0.761
	**42**	3.29	0.85	–**1.18**	1.11	–	–
	**43**	3.36	0.85	–**1.27**	1.11	–	–
	**44**	3.43	0.79	–**1.40**	1.72	–	–
	45	2.92	1.00	−0.77	0.02	0.58	0.770
	**46**	**3.62**	0.75	–**2.00**	**3.34**	–	–
	47	2.87	1.07	−0.76	−0.09	0.55	0.775
	**48**	3.40	0.81	–**1.38**	1.84	–	–
	**49**	**3.65**	0.71	–**2.35**	**5.80**	–	–
	50	3.16	0.87	−0.98	0.72	0.55	0.776
	**51**	**3.89**	**0.40**	–**4.04**	**17.45**	–	–
Father-related adaptation (α = 0.881)	**52**	**3.78**	0.65	–**3.71**	**15.09**	–	–
	**53**	**3.63**	0.76	–**2.56**	**7.31**	–	–
	**54**	**3.57**	0.81	–**2.25**	**5.30**	–	–
	**55**	3.39	0.88	–**1.73**	**3.08**	–	–
	56	2.88	1.11	−0.85	−0.03	0.66	0.865
	**57**	3.30	1.05	–**1.52**	1.54	–	–
	58	2.90	1.12	−0.92	0.09	0.73	0.855
	59	3.09	0.99	−1.03	0.58	0.72	0.858
	**60**	3.32	0.97	–**1.51**	1.81	–	–
	**61**	3.21	1.02	–**1.28**	1.04	–	–
	**62**	3.27	0.95	–**1.42**	1.84	–	–
	63	2.82	1.04	−0.80	0.19	0.72	0.858
	**64**	**3.53**	0.84	–**2.15**	**4.72**	–	–
	**65**	3.36	0.96	–**1.65**	**2.28**	–	–
	**66**	3.33	0.96	–**1.55**	**2.01**	–	–
	**67**	3.32	0.90	–**1.40**	1.73	–	–
	68	2.93	1.06	−0.82	−0.04	0.68	0.863
	69	2.45	1.23	−0.47	−0.77	0.56	0.880
	70	3.02	1.11	−1.07	0.33	0.63	0.869
	**71**	3.41	0.95	–**1.80**	**2.88**	–	–
Personal adaptation (α = 0.875)	**72**	1.82	1.28	0.12	−1.11	**0.31**	**0.875**
	73	2.65	1.31	−0.72	−0.64	0.64	0.860
	74	2.57	1.38	−0.63	−0.88	0.56	0.864
	**75**	**3.62**	0.88	–**2.59**	**6.19**	–	–
	76	2.05	1.45	−0.09	−1.36	0.44	0.869
	77	2.91	1.16	−0.99	0.09	0.60	0.862
	78	2.46	1.42	−0.50	−1.10	0.52	0.865
	79	2.00	1.39	−0.04	−1.26	0.54	0.865
	80	2.08	1.41	−0.07	−1.31	0.51	0.866
	81	2.16	1.34	−0.17	−1.15	0.57	0.863
	82	2.59	1.27	−0.54	−0.79	0.57	0.863
	83	2.62	1.29	−0.59	−0.78	0.52	0.865
	84	2.71	1.15	−0.79	−0.14	0.53	0.865
	85	2.98	1.29	−1.10	0.00	0.53	0.865
	**86**	3.27	1.09	–**1.54**	1.53	–	–
	**87**	3.09	1.14	–**1.29**	0.82	–	–
	88	2.28	1.35	−0.30	−1.12	0.52	0.865
	**89**	3.11	1.19	–**1.27**	0.57	–	–
	90	2.34	1.34	−0.39	−1.05	0.51	0.866

*Items in bold comply with one of the exclusion criteria described in the method section*.

An EFA was conducted with the first half, and Bartlett's sphericity test (χ^2^ = 12473.90; *p* < 0.001) and the Kaiser-Meyer-Olkin (KMO = 0.91) showed satisfactory results. The initial EFA indicated 10 factors with eigenvalues of above 1, although Timmerman and Lorenzo-Seva's (2011) parallel analysis recommended considering five factors. Subsequently, a 5-factor solution was forced that explained 53.02% of the variance. Items that did not comply with the criteria described in the method section were removed. The original 6-factor structure was not replicated, as any item from the original *mother-related adaptation* factor was retained in this sample. We observed acceptable reliability indexes by factor as well as for the global scale (ω = 0.900). The results are summarized in Table 2.

**Table 2:** Summary of information about factors extracted from the EFA.

**Item (extract)**	**Communality**	**F1**	**F2**	**F3**	**F4**	**F5**
		**Peers**	**Teachers**	**School as institution**	**Father**	**Personal**
3. I think I have nice teachers	0.888		0.688		
5. My teachers think highly of me	0.835		0.757		
7. I like my teachers	0.903		0.741		
13. My teachers show interest on me	0.867		0.795		
14. My teachers talk with me	0.906		0.733		
16. My classmates appreciate me	0.868	0.810			
18. My classmates behave well with me	0.940	0.841			
19. My classmates defend me from critiques	0.733	0.698			
20. My classmates support me if I ask for help	0.907	0.742			
21. My classmates speak well of me	0.837	0.777			
25. I think I have nice classmates	0.950	0.753			
26. I am very attentive	0.860			0.843	
28. I comply with school rules even I dislike	1.000			0.668	
29. I do homework	0.883			0.847	
30. I pay attention to teacher's lessons	0.911			0.894	
56. My father comments positively about me	0.722				0.645
58. I like my father's way of thinking	1.000				0.800
59. My father positively values my behavior	0.862				0.748
63. My father likes my ideas	0.822				0.845
68. My father respects my ideas even he dislikes	0.879				0.711
69. My father and I have similar preferences	0.866				0.683
70. My father respects my preferences	0.865				0.686
73. I have problems with myself	0.855					0.686
74. I feel nervous with no reason	0.856					0.694
77. I feel sad and boring	0.724					0.658
78. I feel ashamed if someone criticizes me	0.826					0.651
79. I feel bad when I am wrong	0.802					0.631
80. I have to push myself to do things	0.830					0.633
81. It is difficult for me to make decisions	0.833					0.637
82. I am afraid to difficulties and obstacles	0.983					0.655
85. I find myself as different from others	0.698					0.633
Explained variance		23.99%	12.07%	7.22%	5.58%	4.16%
Inter-factor correlation					
F2 Teachers		-	0.263^**^	0.223^**^	0.322^**^	0.229^**^
F3 School as institution			-	0.528^**^	0.314^**^	0.036
F4 Father				-	0.297^**^	0.130^*^
F5 Personal					-	0.176^**^
Ordinal α		0.897	0.860	0.885	0.889	0.870

* p < 0.005

** *p < 0.001*.

Several CFA analyses were performed with the second half of the sample. Given the significant correlations between the factors, together with issues of theoretical interpretation, we considered a second-order factor called *school adaptation* (integrating the *teacher-related adaptation* and *school-as-an-institution-related adaptation* factors), as well as a third-order factor called *adaptation* (including all the factors). Therefore, a model (M1) including the five factors extracted from the EFA with a second-order factor (*school adaptation*) and a third-order factor (*adaptation*) was computed. Since the EFA excluded the *mother-related adaptation* factor and, by itself, the *father-related adaptation* factor is considered a weak indicator of family adaptation from a theoretical point of view, another model (M2) was tested: M2 was similar to M1, but from which the *father-related adaptation* factor was excluded.

Mardia's coefficient indicated violation of the multivariate normality assumption (Mardia_M1_ = 20.20 and Mardia_M2_ = 21.46), so we used robust estimators. Since the second-order factor consisted of only two first-order factors, identification problems emerged (Bollen, 1989). Therefore, the error variances of both first-order factors were constrained (Rossen et al., 2008). Table 3 shows the goodness-of-fit estimators for M1 and M2.

**Table 3:** Goodness-of-fit indexes of the models resulting from the CFAs.

**Model**	**S-Bχ^2^**	**df**	**NNFI**	**CFI**	**RMSEA**	**90% confidence** **interval RMSEA**
M1	820.29^*^	421	0.93	0.94	0.05	0.04–0.05
M2	515.89^*^	248	0.94	0.95	0.05	0.04–0.05

* p < 0.001.

*M1 = Model 1, that included the 5 factors extracted from the EFA (peers-related, teachers-related, school-as-institution-related, father-related and personal adaptation) with a second-order factor (school adaptation) and a third-order factor (adaptation). M2 = Model 2, this model was similar to M1, except for the father-related adaptation factor, that was excluded*.

M1 and M2 were compared as rival models. For this purpose, we performed the significance test on the difference between Satorra-Bentler scaled chi square statistics (Satorra and Bentler, 2001; Crawford and Henry, 2003), with better goodness-of-fit indicators for M2, as ΔS-Bχ^2^_(173)_ = 306.45, *p* < 0.001. Therefore, following Hair et al's (2008) recommendations, we examined the statistical contribution and regression coefficients of the variables, resulting in *p* < 0.001 and adjusted *R*^2^ values ranging from 0.22 to 0.87. No high coefficient standardized errors were observed (Batista and Coenders, 2000). Second- and third-order factors significantly contributed to M2, although *school-as-an-institution adaptation* and *personal adaptation* showed weak standardized factor coefficients (0.07 and 0.9, respectively). Therefore, M2 was tested again after deleting the third-order factor *adaptation* (this is M2'). Goodness-of-fit indicators remained satisfactory [S-Bχ^2^_(251)_ = 538.08, NNFI = 0.94, CFI = 0.94, RMSEA = 0.05 with a confidence interval of 0.4–0.06], not differing from M2. Consequently, the M2', a second-order four-factor solution made up of 24 items, was the one finally adopted. The reliability of this model was 0.957.

## Discussion

The aim of this study was to provide evidence attesting to the psychometric soundness of the *EMA* (García and Magaz, 1998). This effort goes beyond previous empirical approaches that offered only descriptives for Spanish population and item-level analyses (Aragón and Bosques, 2012; Suárez et al., 2012), inserting psychometric analysis of *EMA* for the first time in international scientific discussion. To this end, both items and factor structure were analyzed. A four-factor solution made up of 24-items was finally adopted, offering a shorter version of the instrument that includes peers-related, teacher-related, school-as-an-institution-related, and personal adaptation. A second-order factor integrated both teacher-related and school-as-an-institution related adaptation. This solution partially replicates the original factor structure. These results are consistent with previous psychometric analyses of other *Magallanes* instruments, which proved psychometric robustness (Servera et al., 2009; Martínez-Monteagudo et al., 2013) but included slight adaptations (Ison and Carrada, 2011).

The factor structure of the *EMA* presented in this paper is consistent with an organizational but non-deterministic view of adolescent development (Cicchetti and Toth, 1997; Wenar and Kerig, 2000), already described in the introduction section. Thus, a moderated inter-factor correlation was observed, but no third-order factor referring to global adaptation emerged. This is, as proposed in the introduction, adjustment at school context in adolescence refers to a multi-domain adjustment that encompasses not only academic, but also individual and social performance (Flammer and Alsaker, 2006; Wentzel and Looney, 2007). As previously stated, this perspective is in consonance with the integrated view of formal education that addresses twenty-one century agenda (Delors et al., 1996; OEI, 2010) that enhances the role of school context to promote social competences as self- and others understanding, as well as understanding of interpersonal interactions and social systems (Hutto et al., 2011). In sum, in our opinion these findings highlight the need to include comprehensive evaluations in the school context for Spanish students, if the aim is to promote adolescent development from an integrated perspective (Pozo and Pérez, 2009).

Despite the relevance of family influences for adolescent adjustment (Dowrick and Crespo, 2005), our results suggest that the *EMA* does not adequately evaluate this area. The majority of mother- and father-related items showed problems at the item-level analysis. In EFA, the mother-related factor was not found; in CFA, the inclusion of the father-related factor in the model showed worse adjustment-of-fit indicators. In our opinion, this scale assesses family adaptation from a dyadic approach; this is, it measures adjustment to family context from a person-to-person perspective (mother-related and father-related adaptation). However, nowadays there is consensus about that family influences are not limited to person-to-person interaction at a dyadic level. The family as a system encompasses complex rules, bonding, and circular interactions that play a role for adolescent development (Olson and Gorall, 2003). It is possible that the underlying dyadic approach in *EMA* does not adequately cover the ecological-systemic functioning of family dynamics (Walsh, 2016). As a result, mother- and father-related adaptation factors did not worked correctly.

The domains remaining in the final proposal for the *EMA* refer to school (both teacher- and institution-related), peers and personal adaptation. For both teacher- and peers-related adaptation, the items related to the frequency of the interaction were dropped out, and those concerning the satisfaction with the relationship and the perceived support remained. This result gives empirical support to the idea that perceived components of social interaction are critical in comparison to structural ones. This assumption is widely recognized in social support arena (Uchino, 2004), and has been explained from social cognition studies, stating that for both teacher- and peers-related adaptation the development of views of what “appropriate” or “expected” in these interactions are necessary (Brizio et al., 2015). However, from our knowledge this has not been tested from a psychometric approach on adolescent adjustment at school. Moreover, items referring to commitment with values and norms remained for the adaptation to school as an institution domain, and those focused on general satisfaction disappeared. This result emphasizes the independent and relevant component of feeling of belonging to the school institution (Kearney, 2008).

In sum, the version of the *EMA* presented in this paper is shorter and covers several core components of adjustment at school in adolescence. As pointed out in the introduction section, we do not count on a single model for this construct in adolescence. However, the facets cover by the *EMA* coincide with the broad categories identified in the most recent papers on adjustment evaluation within the school context in other developmental and educational transition, as adaptation to the college is (Credé and Niehorster, 2012). According to these authors, not only academic, but also social, and personal-emotional adjustment need to be considered in relation to how students adapt to the school environment. Despite the relevance of this, it has been discussed here that the *EMA* instrument does not cover adequately family adaptation. Moreover, it should be noticed that other individual components that influence school adjustment are not included in this measure, as expectations, goal orientations or self-concept (Bruyn et al., 2003; Dowrick and Crespo, 2005; Anderman and Kaplan, 2008).

Despite the aforementioned limitations, in our opinion, the short version of the *EMA* proposed in this paper constitutes a useful tool to evaluate adolescent adjustment for diverse purposes. School teachers are in a privileged position to enhance adolescents' health and development. For this aim, the *EMA* constitutes an interesting screening tool to identify vulnerable domains of adjustment at school that may need to be promoted, besides academic achievement. Moreover, the *EMA* can provide a holistic view of adolescent adjustment at school either for initial evaluations or as a progress indicator. This version of *EMA* is a context-based, reliable, and cost-effective measure of adolescent adjustment at school context from a developmental perspective respectful with organizational, contextualist, and positive principles. As designed and tested with Spanish-speaking adolescents, the cultural validity of this measure is also a strength. Nevertheless, the short version of the *EMA* proposed in this paper is limited as no empirical evidence on its relationship with other related constructs is provided. Thus, the incoming challenge is to probe whether this shorter version works better than the full-length version. Moreover, invariance testing accordance with adolescents' sociodemographic and psychoeducational profiles should be examined in future research. These advances will serve as the starting point to disseminate the *EMA* among school teachers so that they can consider this tool as a valuable resource to assess adolescent adjustment.

## Author contributions

All the authors have made substantial contributions to the conception of the work, as well as the analysis and interpretation of its data. All the authors have reviewed the work and have given their final approval of the version to be published. All authors agree to be accountable for the content of the work contributed to the manuscript and agree with this submission.

### Conflict of interest statement

The authors declare that the research was conducted in the absence of any commercial or financial relationships that could be construed as a potential conflict of interest.
